# How Soon Hath Time… A History of Two “Seminal” Publications

**DOI:** 10.3390/cells10020287

**Published:** 2021-02-01

**Authors:** Geoff A. Parker

**Affiliations:** Department of Evolution, Ecology and Behaviour, University of Liverpool, Biosciences Building, Crown Street, Liverpool L69 7ZB, UK; gap@liverpool.ac.uk

**Keywords:** sperm competition, post-copulatory sexual selection, origin of sexes

## Abstract

This review documents the history of the two papers written half a century ago that relate to this special issue of *Cells*. The first, “Sperm competition and its evolutionary consequences in the insects” (*Biological Reviews*, 1970), stressed that sexual selection continues after ejaculation, resulting in many adaptations (e.g., postcopulatory guarding phases, copulatory plugs, seminal fluid components that modify female reproduction, and optimal ejaculation strategies), an aspect not considered by Darwin in his classic treatise of 1871. Sperm competition has subsequently been studied in many taxa, and post-copulatory sexual selection is now considered an important sequel to Darwinian pre-copulatory sexual selection. The second, “The origin and evolution of gamete dimorphism and the male-female phenomenon” (*Journal of Theoretical Biology*, 1972) showed how selection, based on gamete competition between individuals, can give rise to anisogamy in an isogamous broadcast spawning ancestor. This theory, which has subsequently been developed in various ways, is argued to form the most powerful explanation of why there are two sexes in most multicellular organisms. Together, the two papers have influenced our general understanding of the evolutionary differentiation of the two forms of gametic cells, and the divergence of sexual strategies between males and females under sexual selection.

## 1. Introduction

At the request of the Editors of this *Cells* special issue, I attempt here to give a history of two early “seminal” papers (a pun that has been used several times). The first, “Sperm competition and its evolutionary consequences in the insects” [1], my 1970 *Biological Reviews* article, has been credited as the catalyst for the now large field of post-copulatory sexual selection [2,3], i.e., sexual selection that continues after ejaculation. The second, less cited paper on “The origin and evolution of gamete dimorphism and the male-female phenomenon” [4], with co-authors R. R. Baker and V. G. F. Smith, appeared in the *Journal of Theoretical Biology* in 1972, and has had a role in establishing a theoretical foundation for the evolution of the gametic cells and the two sexes.

Precisely how these two papers germinated can ultimately be traced back to an early and enduring passion for natural history and animals, which led me eventually to my Ph.D. research at Bristol University on sexual selection in the yellow dung fly, *Scathophaga* (=*Scatophaga*) *stercoraria* L. This rather squalid topic developed out of my final-year undergraduate project; my somewhat precarious pathway to that destination has been documented elsewhere [5,6]. However, my postgraduate research on dung flies undoubtedly played a key role in generating the questions and ideas that led to those two papers.

## 2. From Dung Flies to Sperm Competition, Anisogamy—And Other Things

As a final-year undergraduate and postgraduate at the University of Bristol, I benefitted immensely from discussions with Robin Baker, a student contemporary in Zoology at Bristol University, 1962–1968. Rob’s Ph.D. research concerned the evolution of butterfly migration, while I worked on sexual selection in dung flies. Rob was a key influence, a sharp, lateral-thinking intellect. We became preoccupied with the mechanism of natural selection, and how selection acting on individuals (individual selection) often failed to fit plausibly with adaptive functions then popular in behaviour and ecology. Our worries echoed those of the great G. C. Williams, whose key publication [7] we were unaware of at the time [5,8]. As our confidence in the individual selection approach grew, I can remember our initial horror when another postgraduate directed us to V. C. Wynne-Edwards’ book [9], which proposed group selection (selection acting at the level of groups) as a validation of the “advantage to the species” shorthand we had come to distrust so much. The horror was short-lived, and I cannot capture better the excitement of that formative era in the 1960s than Rob’s own powerful account [10] of that time:

Geoff Parker and I were very different characters, yet around 1964 we both found a lifetime’s inspiration from a single controversy. Outside of Bristol a war was raging in the world of evolutionary theory, triggered by V. C. Wynne-Edwards’ 1962 book extolling the power of group selection to bring about evolutionary change. People were taking sides, even in Bristol, with Geoff and I aligning with what seemed to be the minority at the time, the Individual Selectionists. In a bar or a refectory we would defend our cause against all-comers. We would even go to the then-extreme of claiming individual selection to be a force in the evolution of behavior. It was our first experience of the buzz to be gained from the defending of an academic idea that, apparently, was outrageous. At a time when few believed that behavior of any sort was even heritable, our backs were against the wall for much of the time.

In fact, the battle was with the ethologists and ecologists, who at that time typically avoided details about how selection worked, while evolutionary biologists and population geneticists were generally not impressed with group selection. The debate catalysed and generated Richard Dawkins’ celebrated discourse, *The Selfish Gene* [11], and since then, it has been through various convulsions. It is now recognized that group selection can operate if not too strongly opposed by individual selection; the current view is that it is a form of kin selection (selection that accounts for a gene’s action both on the individual actor and on the same gene in relatives affected by that action) and can be analysed in such terms (e.g., [12,13,14]). Rob Baker became a leading authority on animal migration [15], and later made a major research transition, which was to pioneer in 1995 the field of the evolutionary psychology of human sperm competition with Mark Bellis [16] (for Rob’s absorbing narrative of how this came about, see [17]), after which he left academia to pursue a very successful career as a writer and novelist (often themed around his work in evolutionary psychology [18,19]), and has recently returned to science.

My Ph.D. project on dung flies began as a final-year undergraduate project [5,6]. The male behaviour undoubtedly led me to think about sperm competition (strictly, inter-ejaculate competition, i.e., competition between ejaculates from different males) and its evolutionary consequences, which in turn generated an interest in the evolution of anisogamy (gametes of two different sizes in a given species), the key to the question of why there are so ubiquitously two sexes. Rob Baker again gives a very vivid account [10]:

…we shared a freezing apartment on the outskirts of Bristol. There, on those nights that we were both in, we would sit bent over the single gas fire, singeing gas-tainted toast on long forks and talking about sex—as postgraduates do. Unlike most postgraduates, our conversations revolved around dung-flies and Geoff’s ground-breaking notion of sperm competition. It is fun to think that for those few months that fire-lit room in Bristol could have been the only place in the world where sperm competition was being discussed…Or—another favorite sexual topic because it seemed so impenetrable—we would agonise over why there were two sexes, and only two. That question really stumped us, maybe even shook our faith a little. How could such a question possibly be answered by individual selection?

**Figure 1:**
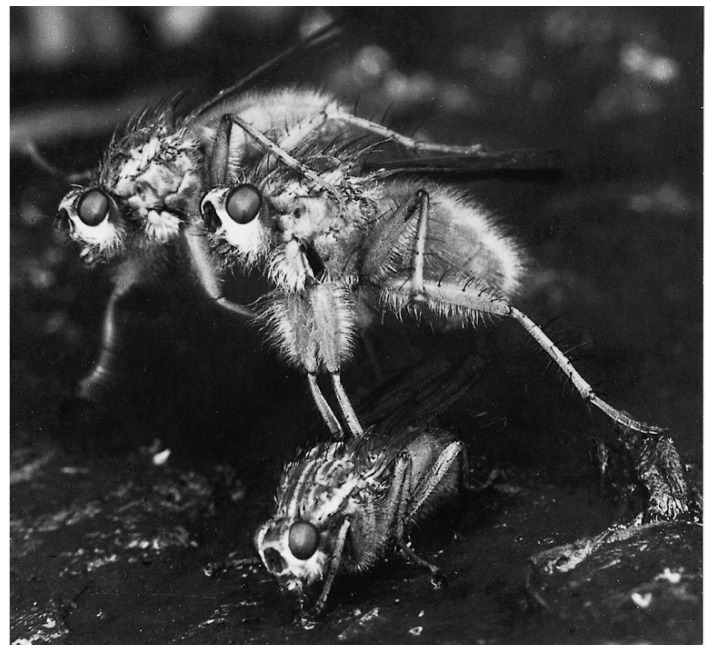
A male dung fly guarding the female he has just mated with against an attacker while she lays her eggs in the cattle dropping. Photograph is from my Ph.D. thesis in 1968, published in 1970 [25].

The dung flies also led me to ponder about other general questions in behavioural and evolutionary ecology [20], emerging sciences at the intersection of evolution with behavior and ecology, such as mate-searching and animal distributions [21,22], sexual conflict [23], and the rules governing animal fighting behaviour [24]. Males compete intensely to mate with gravid females as they arrive to lay their eggs in fresh cattle dung. However, one aspect of the male’s behaviour is unusual: after mating, the male guards the female while she lays her eggs (Figure 1). It was this that made me think about sperm competition. Males seemed to be guarding their paternity—searching males made fierce attempts to take over an ovipositing female, and the guarding male used various specialised responses that (usually) prevented this [25]. If these failed, the new male mated with the female before she resumed oviposition, guarded by the new male. I realised that gaining matings was not the end of the story—ultimately, sexual selection depends on gaining fertilisations.

An important finding from my early dung fly research—predominantly a field study—was that, for a male, the fitness value of time could be expressed as the expected number of fertilizations achieved per minute of reproductive activity (in my study population, 0.23 eggs/male/min. [21]). A given male’s fitness also depends on the strategies of other males. Thus, to quantify selection on guarding behaviour, I needed to understand how sperm competition operated. As genetic markers were unavailable, I began experiments (completed later in Liverpool) that mimicked the sterile male release technique. I irradiated males using a Cobalt-60 source to “label” their sperm by inducing dominant lethal mutations; eggs were fertilised by irradiated sperm, but did not hatch. A male’s fertilisation gains increased with his copula duration, and on average, the last male to mate fertilized over 80% of the eggs remaining to be laid [26]. I calculated that the guarding behaviour (Figure 1) would be strongly favoured by post-copulatory sexual selection [27]. Most females arriving at the droppings already contain sperm from previous matings [28]. Coupled with the Cobalt-60 experiments, I could also estimate the optimal copula duration for a male. The observed duration was in the general area predicted—the first evidence that sexual selection had shaped sperm allocation in a species. A more extensive account of the contribution of the dung fly to the study of sperm competition is given elsewhere [29].

## 3. Sexual Selection and the Behavioural Ecology Revolution of the 1970s

By 1965, when I began my Ph.D., Darwinian sexual selection [30] had become unfashionable, with a few rare exceptions (e.g., [7,31,32]). I was extremely lucky to begin research around the beginning of what was to become the “behavioural ecology revolution” of the 1970s [33], and feel very privileged to have played some part in that development. This “revolution” effectively displaced the (usually implicit) group or species selectionism prevalent at that time in ethology and ecology, with arguments based on selection acting on individuals [7] (as Darwin had originally proposed [30,34]); it replaced much of ethology with a selection-based approach to behavioural adaptation, characterised by an awareness of conflicts of interest between individuals and the use of evolutionary modelling to understand adaptation [33,35,36].

After my Ph.D., in late 1968 I began work as Assistant Lecturer in Zoology in Liverpool University, and in autumn 1969 wrote a course of four lectures (the notes still survive) on sexual selection for final-year students. The first includes a discussion of Bateman’s (now classic) 1948 paper [31] and the evolution of the sex ratio:

Interesting to note that the 1:1 sex ratio…conflicts with the “best advantage to the species” philosophy. If…more females were produced, then reprod. [reproductive] rate of the species would increase.

**Figure 2:**
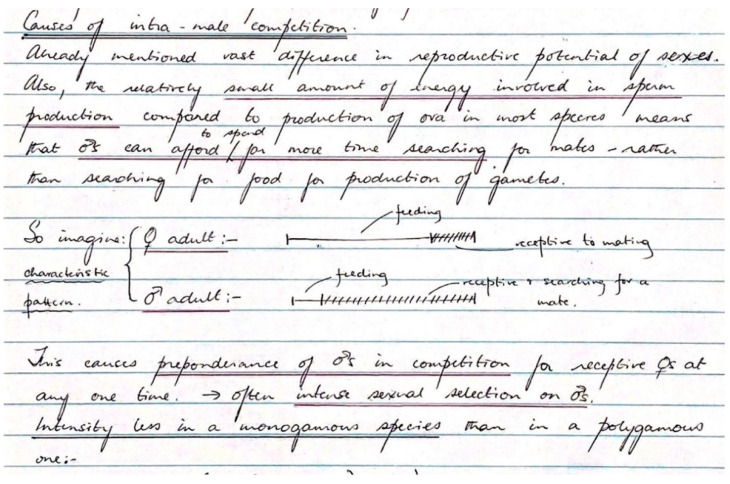
Inset from the first of my four lectures on sexual selection to final-year students, late 1969.

Time budgets were very much on my mind because of the dung fly work, and a section entitled “Causes of intra-male competition” includes a diagram (Figure 2) with two lines (i.e., male, female) of equal length that foreshadows the “time in”, “time out” approach to sexual selection (see e.g., [37]). With a 1:1 sex ratio, the length of each line represented the time between clutches for a female, and the unshaded areas on each line represented “time out” of the mating arena due to feeding, etc., which is much greater for females. The remaining shaded areas represented “time in”—i.e., time searching/competing/receptive for mates. I argued (Figure 2) that, since in mobile animals, an ejaculate generally costs far less time out than producing a clutch of eggs/offspring, males are likely to be under more intense competition for matings than females:

This causes preponderance of males in competition for receptive females at any one time… [generating] often intense sexual selection on males.

Several of the dung fly papers were published in 1970. I had corresponded with Bob Trivers, who had read them while writing his classic 1972 paper on sexual selection and parental investment [38], which he had sent to me in manuscript for comments. I had loved it, and wrote back (3 July 1971):

May I say immediately that I have been extremely impressed with your paper on sexual selection…to my mind it is easily the most important contribution to the subject since Bateman’s work.

He replied (25 July 1971) that he was delighted that I liked the paper, that my letter had spurred him to work on it some more, and went on to state:

Your papers measuring opposing selection pressures for such traits as the passive [i.e., guarding] phase of males are a pleasure to read. As you know it’s extremely rare to find such measurements of opposing selection pressures. Someday, particularly for social traits, we will have to work out some more formal principles for applying natural selection than are commonly employed: you routinely think in terms of selection pressures operating simultaneously on several individuals at the same time, but this is not common, and it should be of value someday for someone to formulate in detail working rules by which one makes sophisticated functional arguments.

Remarkably, Bob Trivers was foreshadowing the development of the evolutionarily stable strategy (ESS) concept, pioneered just two years later by Maynard Smith and Price [39], and which was to play a large part in the behavioural ecology revolution (see [33]).

Before the 1970s, sexual selection attracted only muted attention from ethologists, and was often met with criticism and skepticism [40]. Interpretations were usually based on natural selection, possibly because of the prevailing (often implicit) group/species selectionism of the time, and perhaps also because Darwin’s original definition (the advantage which certain individuals have over others of the same sex and species solely in respect of reproduction) led to disqualification of any behaviour resulting in a general increase in population mean fitness. In 1889, Wallace [41] was highly critical, dismissing female choice, and seeing male–male competition as entirely the product of natural selection (roughly, stronger males produce stronger offspring that survive better). Fisher in 1930 [42] proposed the now-influential “runaway” theory for female choice (the accelerating spread of a male trait gene with a gene for female preference for the trait). This was investigated formally by his last student, Peter O’Donald, in 1962 [43], but these works appeared not to permeate into mainstream ethology and ecology until the 1970s. Huxley had published two notable reviews in 1938 [44,45], which also dismissed female choice from sexual selection, ascribing it to “epigamic selection”, serving to facilitate mating efficiency, hence natural selection. He did, however, acknowledge intra-male competition, arguing that the balance of selective advantages arising via sexual and natural selection components varies greatly. One of Huxley’s reviews [45] began with the comment that none of Darwin’s theories have been so heavily attacked as that of sexual selection, something that continues even today (e.g., [46]). His long monograph, *Evolution: the Modern Synthesis*, in 1942 [47] devoted only about half a page (of almost 600) to the subject, and it was not even included in the topic index. Finally, as late as 1972, the prominent evolutionary biologist Ernst Mayr, although giving limited recognition to sexual selection, consigned intra-male competition to natural selection [48]. The catalyst for the sudden explosion in sexual selection studies which began in the 1970s was undoubtedly Bob Trivers’ [38] classic article, published (ironically) in the same monograph as Mayr’s. By the time of Malte Andersson’s excellent monograph in 1994 [40] covering mainly pre-ejaculatory sexual selection, the acceptance, interest, and the literature on the topic were completely transformed.

## 4. Sperm Competition

### 4.1. Writing and Publication

My ponderings about sperm competition and its evolutionary consequences continued after arriving at Liverpool in late 1968. I had a very large teaching load in my first two years, and what little free time left was spent writing papers from my thesis and completing the Cobalt-60 experiments. By late 1969–early 1970 I had begun work on “seminal” paper number one, “Sperm competition and its evolutionary consequences in the insects” [1]. In my 1969 sexual selection course (see above), the first lecture ended by claiming that sexually-selected characters may be pre-, syn-, or postcopulatory (with definitions), noting that “…though virtually all attention is given to precopulatory adaptations.”

In addition, close to the beginning of the last lecture, on postcopulatory adaptations was this:

So far, [it] has been assumed that sexual selection concludes with the act of mating. This is an oversimplification…A better measure of sexual selection advantage is the fertilization rate in terms of the no. of offspring fertilized by a given male/unit time rather than no. of females inseminated/unit time.

Shortly after came an early definition:

Sperm competition may be defined as the competition, within a female, of the sperm from two or more males over the fertilization of that female’s ova.

This was followed by a short discussion of plugs and guarding phases in vertebrates, and a much longer account of sperm competition and its resulting adaptations in invertebrates, often featuring dung flies.

Writing the sperm competition review was relatively fast for me at that time, possibly because I had given so much previous thought to the topic. In those non-digital days, writing a paper was a much more labour-intensive task than now, involving much library time, delays waiting for “inter-library loans”, and typing and correcting manuscripts and their carbon copies.

Fortunately, I filed away all correspondence relating to my publications. The first draft of the manuscript was received at the *Biological Reviews* editorial office on 18 May 1970. The editor was then the polymath E. N. Wilmer FRS, histologist, biologist, artist, and garden designer. I retained a handwritten draft of my submission letter (which I would have then retyped for inclusion with the manuscript), giving my rationale for the paper:

I have attempted to analyse the intra-sexual selective pressures which arise in the insects as a result of the high level of sperm competition to which the group appears preadapted. Many copulatory and post-copulatory adaptations seem explicable in terms of this analysis. Previous considerations of sexual selection have tended to concentrate on pre-copulatory adaptations involving mechanisms by which males increase their chances of mating. I hope that the present review concerning sexual selection during and after mating will serve to link experimental genetics with the field behaviour approach.

By “appears preadapted”, I alluded to the presence of sperm storage organs in the female, in which sperm from different males could compete.

A neat, handwritten letter dated 25 June from the editor (from his “holiday retreat” in Llanidloes, Mid Wales) states that he had “very much enjoyed” reading the review, which he found “most interesting”. He had received comments from the referee: they both agreed that it should appear in *Biological Reviews*, but that it was too long (estimated at c. 25,000 words). He suggested that it could be improved:

…by bringing out the general principles perhaps more clearly…The treatment as it stands would be more suitable for a book…Will you please, without delay, see what you can do to comply with these ideas, and return the MS to me in Cambridge as soon as you possibly can. If we can get it to the printers in mid-July, there is a good chance that it will appear in the September number.

He requested that I carefully check references in the text and the list, and that they conform with journal practice, and ended with:

I hope you can do all this rather troublesome work without undue disruption—But I’m quite sure the referee is right in his comments, and in any case we are trying now to keep to the 20,000 word limit, because there are a very large number of reviews promised and space will have to be conserved.

It was the exam and holiday season, but I completed the revision, and a second, rough, handwritten letter—again a draft of what I must have retyped to accompany the revised manuscript—states that I had removed much of the details of experimental evidence for sperm competition (some 3000 words) and a further 1500 words from the rest of the text, which then remained at around 20,000 words. I mentioned that the decimated experimental evidence section had been retyped, but wrote:

I hope you will forgive me for not having retyped the rest…As time is limited I felt that these measures might be inadvisable.

The proofs appear to have arrived in mid-August, and I have a handwritten letter from E. N. Willmer (17 August), noting:

I am thinking of making alterations and corrections as indicated on the enclosed sheet, and would like to have your blessing or veto.

He suggested that I should send the corrections to his holiday home, where he would be staying the following week. I seem to have agreed to almost all these alterations, and received a letter from him (23 August) mentioning that my final corrections had been received and that the paper had gone to the press. I shall always be deeply indebted to him for the time and care he spent on this manuscript, and for his very polite and gentlemanly handling of it.

The review was published on 1 November, 1970, 6.5 months after submission, which was fast; at that time, papers commonly took well over a year from submission to publication. It contained two early citations of Bateman’s classic 1948 paper [31], which (then) was cited only around once a year, but is now much cited as a key focus in sexual selection logic. My citation of Bateman has been criticised recently by Hoquet [49], I believe unfairly (see Supplementary File S1).

### 4.2. Subsequent Developments

**Figure 3:**
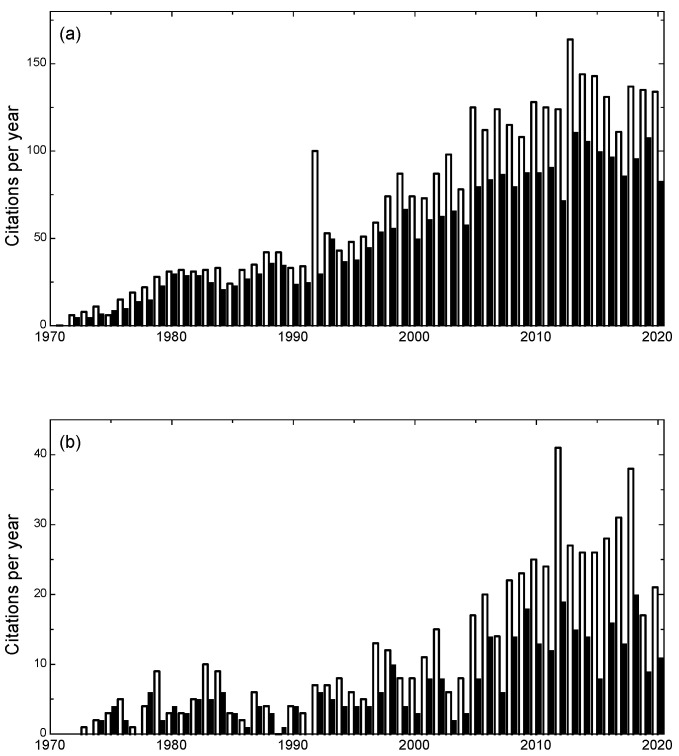
Citations per year of (**a**) the 1970 *Biological Reviews* article on sperm competition, and (**b**) the 1972 *Journal of Theoretical Biology* article on gamete dimorphism and the two sexes. Sources: Web of Science (filled bars), Google Scholar (open bars). Years before 1981 for Google Scholar were completed using the “custom range” facility. Data for 2020 is up to mid-December.

The review attracted very little attention initially (Figure 3a), and few other publications on sperm competition appeared for almost 10 years [3]. In 1975, we published on sperm competition in locusts [50], but my research had mostly diverted to other projects. Things began to change in 1979, which saw a sudden upsurge of interest in America, with the publication of Jon Waage’s [51] demonstration that damselfly males remove rival sperm before ejaculating, Bob Smith’s [52] study showing that male giant waterbugs copulate repeatedly with ovipositing females to ensure paternity of eggs as they are placed and brooded on the male’s back, and the papers of John Sivinski [53] and James Lloyd [54] at an *Insect Behavioral Ecology* symposium of the Florida Entomological Society. The first international symposium on sperm competition was organised by Bob Smith in Tucson in 1980, resulting in the first monograph on the topic in 1984 [55]. Between the late 1970s and mid 1980s, the concept and evidence of cryptic female choice (sperm selection by females) was launched [54,56,57,58], and later established as a field in its own right in 1996 by Bill Eberhard in his classic monograph [59]. Thus, the two components of Darwinian pre-ejaculatory sexual selection, male–male competition and female choice, were joined by the two parallel post-ejaculatory components, sperm competition and sperm selection.

By 1980, though I enjoyed field work and the dung fly project, my interests had become focused on theory, and I published some early models (1982) of how sperm competition affects sperm allocation, in a paper mainly focused on the maintenance of anisogamy [60]. Ric Charnov had published (1980) on how “local mate competition” (related to what I term “sperm competition level”) would affect allocation to male and female “function” in simultaneous hermaphrodites (see also [61]), and in 1990, I began analysing “sperm competition games” in earnest [62,63]. After that, many variants on these games (mainly differing in conditions faced by a male at the time of ejaculation) were analysed by myself and others, so that over the next 20 years a substantial theory base became established (reviewed in [64,65]). This stimulated empirical tests, and publications in sperm competition steadily increased over that period, levelling off around 2008 [3]. Citations of the 1970 review continued to increase up to around 2015, but now also seem to have levelled off (Figure 3a). Perhaps the major driver of research interest was the establishment of the biennial *Biology of Spermatozoa* conference beginning in 1992, pioneered by Tim Birkhead (leading researcher in bird sperm biology and author of monographs on the topic [66,67,68]) and his reproductive biologist colleague at Sheffield, Harry Moore. Several further books on sperm competition have appeared since Bob Smith’s 1984 volume: Leigh Simmons’ excellent survey of the insects in 2001 [69] used the same title as my 1970 review, and showed the remarkable developments in that taxon in the subsequent three decades.

Over the last 50 years, many defensive adaptations to sperm competition shown in 1970 in insects (e.g., copulatory plugs, post-ejaculatory mate guarding) have also been demonstrated in other taxa. However, the major advances have been in adaptations relating to ejaculate production and delivery. Understanding why sperm are small and numerous is no longer difficult [60,70], and theory has been successful in predicting relative testes size [71,72,73] and sperm economics [74,75], i.e., sperm numbers allocated under different biological conditions. Major advances have occurred in our understanding of how sperm competition influences seminal fluid proteins, and their manipulation of female reproductive biology [69,76,77,78,79,80]. One aspect not covered in the 1970 review was how sperm competition may modify sperm cell form and function, something proposed by John Sivinski at the influential meeting of the Florida Entomological Society in 1979 [53]. This has proven more elusive, though great strides have been—and continue to be—made (e.g., [81,82,83,84] and edited volume [68]). Much can be gained from considering the changes in sperm competition level during evolutionary time (the “sexual cascade” [85]), from initially very high levels in ancestral sedentary broadcast spawning invertebrates, to typically lower levels in mobile internal fertilizing species with high pre-ejaculatory male–male competition, generating notable reductions in relative testes size [85,86].

A more detailed timeline of key developments in sperm competition and sperm selection is given by Simmons and Wedell [3], and I give a brief review of the current status of conceptual areas in sperm competition in the same theme issue [70].

## 5. The Evolution of Gamete Dimorphism and Two Sexes: “PBS”

### 5.1. Writing and Publication

The paper on the evolution of anisogamy (or gamete dimorphism, as we called it) was also linked to dung flies and sperm competition: thinking about ejaculates competing posed the question of why male and female gametes, the feature which defines the two sexes, are so different. The paper also had its origin in discussions with fellow postgraduate Rob Baker (see above), but active research on the topic began a year or two later, after Rob had moved from Bristol to Newcastle University. He wrote [10]:

In a single memorable phone conversation with Geoff Parker, by then at Liverpool, I discovered that we had both dreamed-up a solution to our long-standing niggle at the conundrum of two sexes. The answer, we had both decided, had little to do with maleness and femaleness and everything to do with eggs and sperm. Explain the evolution of anisogamy, we encouraged each other, and everything else would fall into place.

Unbeknown to us then, forays into this subject had been made previously, as early as 1932 by Kalmus [87] (see also [88]), and later by Scudo in 1967 [89]. However, these were based on the group/species selection notion that selection would maximise the number of fusions for the entire population. That certainly did not suit our individual selectionist convictions. I tried some initial calculations in which selection acted on individual parents: each parent had the same amount of resource for gametes, but divided it into varying numbers of gametes in a size–number trade-off; these were broadcast and fused randomly with the other gametes spawned simultaneously in a large population. However, initially (see [5]) I did not include enough gamete sizes to allow anisogamy to be generated; the result was directional selection in one direction or another—we wanted disruptive selection between gametes competing for fusions to generate coexisting large and small gametes as a stable polymorphism. That this result might arise if I increased the size range was suggested by Vic Smith, a friend from the Bristol days, then completing his Ph.D. in Liverpool. Rob proposed the initial programming for the first computer simulation, which I modified in various ways and ran at Liverpool. Depending on how a zygote’s size (the sum of the mass of the two fusing gametes) related to its subsequent fitness, it could generate anisogamy (two sexes) from isogamy (one sex; i.e., a population producing one gamete size), or remain at isogamy. I wrote the paper and was excited—this seemed to reveal an answer to one of the biggest questions in evolutionary biology: why are there males and females?

Attempts to discuss the project with Liverpool colleagues were disappointing. I suspect they thought it was largely a non-starter: sex was usually determined by chromosomes; for instance, the mammalian XY and XX chromosome system generated males that produced sperm and females that produced ova—that was a fact of life, why ponder further? I had little feedback from colleagues who saw early drafts of the manuscript (see [5]), but nevertheless proceeded undaunted. The submitted version was received at the London editorial office of *Journal of Theoretical Biology* on 19th February 1972. A letter dated 20 March 1972, signed by the London editor Lewis Wolpert, simply states that:

Our referees have now returned the manuscript by you, Dr. Baker and Dr. Smith, which we are happy to accept. The paper has now been sent for publication.

That is rather unlikely to happen today.

**Figure 4:**
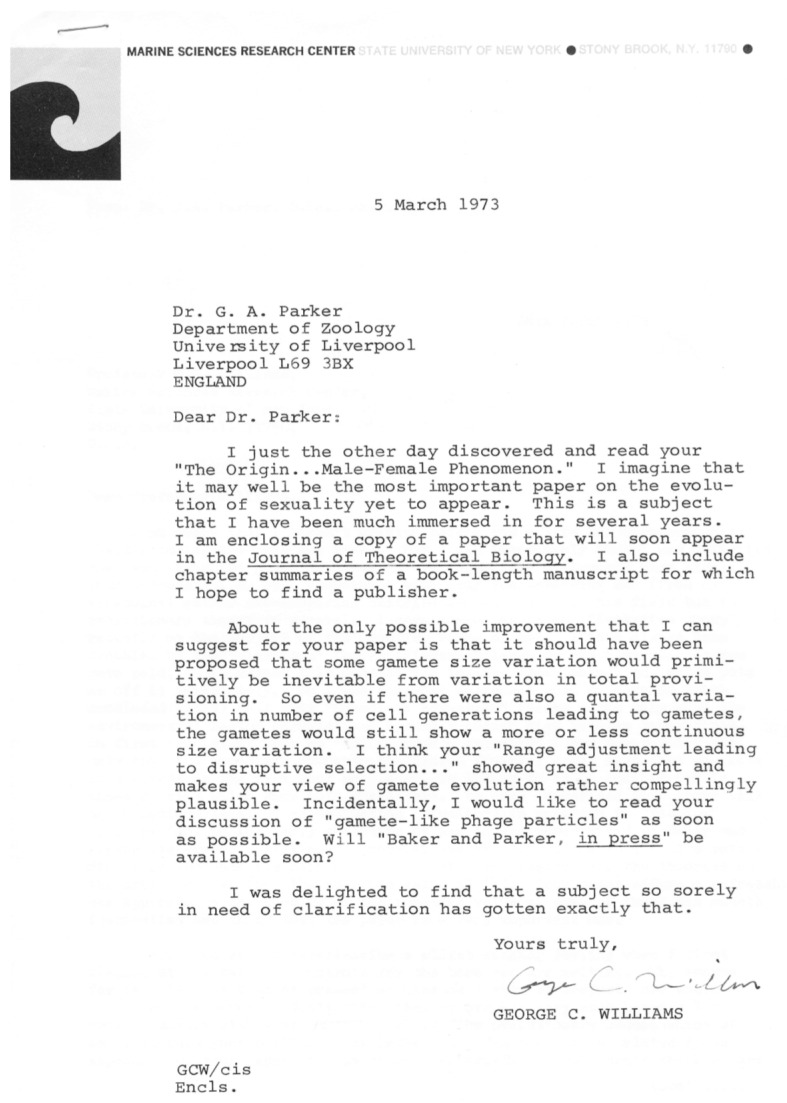
The 1973 letter from George Williams.

Early the following year, I received a very positive letter about the article from George Williams (Figure 4), something which I still greatly treasure. The paper he mentions in *Journal of Theoretical Biology* (see Figure 4) with J. B. Mitton was on the evolution of sexual reproduction [90]. His book (*Sex and Evolution* [91]) appeared in 1975 and gave our paper very favourable coverage. He was quite right to point out that we could have made a good argument that some size variation would be inevitable from variation in total provisioning, and that this would tend to generate some degree of continuous gamete size variation even when the main source of variation was in cell divisions. The other paper that he requested (in press [92]) owed much more to Rob’s efforts than to mine. Despite initially receiving little interest, at least as judged by citations (see Figure 3b), our anisogamy paper gradually gained acceptance as a leading theory for the origin of two sexes [93]. George Williams’ book [91] probably helped, and it was gratifying that two other academic heroes of mine, John Maynard Smith (who later refined our analysis [94,95]) and Ric Charnov both expressed admiration for our theory.

### 5.2. Subsequent Developments

Our 1972 simulation model assumed a large, synchronously spawning population with random fusion between all broadcast gametes, irrespective of size. It was essentially a model of gamete competition, in which proto-ovum producers prospered because their zygotes survived well, and “parasitic” proto-sperm producers flourished by releasing many tiny gametes that claimed most proto-ova. In 1978, John Maynard Smith developed a graphical version [94,95], Graham Bell an analytical version [96], and Brian Charlesworth a population genetics approach, in which he also examined anisogamy evolution via mating types [97]. All supported our 1972 simulations. I had become interested in the evolution of disassortative fusions between large and small gametes during the evolution of anisogamy from isogamy, and published an explanation in 1978 [98] (this paper, “lost” in the editorial office for a year between acceptance and being sent to press, incorrectly cites our previous paper [4] as 1971, not 1972). It was reassuring to know that our theory was not sensitive to the timing of evolution of mating types. Since then, almost all developments have assumed the prior existence of mating types. In revisiting the topic [98], I noticed an error in our simulations. Being poor at programming arrays, I had programmed all the probabilities of fusion between gamete sizes “longhand”, and failed to ensure that those between unequal-sized gametes were twice those of equal-sized gametes. Fortunately, re-runs of our simulations indicated that correcting this error resulted only in small quantitative changes in predictions, and no qualitative differences (see [98]).

Citations remained low (Figure 3b), with occasional further theoretical refinements (e.g., [99]), until a strong criticism by Randerson and Hurst in 2001 [100]. Partly based on flawed mathematics [101], their critique nevertheless stimulated the eminent mathematician Michael Bulmer and I to derive a more complete explanation of the conditions generating the alternative stable states of isogamy and anisogamy [102]. Our model became referred to as “PBS”, and citations rose (Figure 3b), further stimulated by the important monograph on anisogamy edited by Togashi and Cox in 2011 [103], in which my chapter reviewed PBS status and the evolution of gamete cell sizes [104].

Don Levitan, as early as 1996, had pointed out that sperm limitation (where sperm density is too low to ensure high fertilization probability) can be important in broadcast spawners, and is likely to affect anisogamy by increasing the ovum “target” size [105]. In 2008–2010, two theoretical papers resurrected Kalmus’ earlier idea that anisogamy arose through gamete limitation, i.e., to increase fusion probability, but using an individual selection approach [106,107]. The most important recent contribution to the subject was made in 2011 by Jussi Lehtonen and Hanna Kokko [108], who combined gamete competition and gamete limitation into a single rational model. This used most of the PBS assumptions (broadcast spawning, gamete size–number trade-off, zygote size–fitness relationship), but simply varied the degree of gamete competition and gamete limitation, and showed that anisogamy can evolve either under gamete competition, or under strong gamete limitation, provided gamete competition is weak. Gamete competition is nevertheless likely to be the stronger selective force promoting anisogamy unless gamete limitation is very weak or absent [109]. Currently, the coupling of these two components into one “gamete dynamics” model is being employed to demonstrate the remarkable robustness of this approach in explaining anisogamy evolution, for instance when the ancestral isogamous state involves some form of internal fertilization [110].

In PBS, we had suggested a reason why anisogamy should be associated with organismal complexity and multicellularity (a formal basis for this proposition was to follow much later [102]). In 1974, Nancy Knowlton produced evidence that the degree of anisogamy correlates with colony size (number of cells) in volvocine algae, and the volvocines continue to be the primary model group for empirical studies on the evolution of anisogamy [111,112,113]. Graham Bell’s wider surveys in 1978 and 1982 across algal and protozoan taxa offered broad support, though the predicted correlation between the degree of anisogamy and organismal complexity is less distinct across other groups of algae [96,114]. Some anomalies exist, and current efforts seek to explain why this is so [110,115].

## 6. Final Thoughts

When I think back to myself as an academically rather lonely young man of 25 writing the *Biological Reviews* paper in early 1970, a year or so into my post at Liverpool, I would not have believed that it could have led to the developments that were to follow over the next five decades, and that half a century later, it would become one of the papers listed in *Biological Reviews 200^th^ Anniversary Collection* [116] (representing the journal’s highlights, 1820–2020), the focus of a virtual issue of *Behavioural Ecology* [117], a theme issue in *Philosophical Transactions of the Royal Society B* [118], and this special issue in *Cells* [119]. Then, I even had doubts as to how useful the paper would be, despite having thought about it for many years beforehand. In those days, the staff in Zoology all had different research interests to cover the teaching; I had no one at Liverpool to discuss it with. When I rang Philip Shephard (then Head of Genetics at Liverpool) to ask about a paper of his on butterflies, I outlined why I was writing the review (it was largely finished, and I was chasing up remaining references). He made an incisive comment relating to the fact that sperm competition should result in what have become described as “offence” and “defence” adaptations [53,70]. I replied weakly that the entire paper was devoted to developing those concepts. After I put down the phone, I really did wonder whether it was all was too obvious; had I been at the start rather than the end of writing, I may well have not continued [5]. Nevertheless, I suspect that we would probably be at much the same point now—others would have taken the initiative. That is the way with science; perhaps we give too much credit for having the luck to be first. All that a scientist should ever wish is to understand the natural world a little better. It is nevertheless extremely gratifying to feel that one’s scientific colleagues regard one’s contributions as having led to useful developments, and I thank the editors very sincerely for this special issue and the invitation to write this article.

## Data Availability

Not applicable.

## Supplementary Materials

The following is available online at https://www.mdpi.com/2073-4409/10/2/287/s1 Title: Supplementary File S1: Hoquet’s critique of Bateman citation in the 1970 paper.

## References

[1] Parker G.A. (1970). Sperm competition and its evolutionary consequences in the insects. Biol. Rev..

[2] Birkhead T.R. (2010). How stupid not to have thought of that: Post-copulatory sexual selection. J. Zool..

[3] Simmons L.W., Wedell N. (2020). Fifty years of sperm competition: The structure of a scientific revolution. Philos. Trans. R. Soc. Lond. B Biol. Sci..

[4] Parker G.A., Baker R.R., Smith V.G.F. (1972). The origin and evolution of gamete dimorphism and the male-female phenomenon. J. Theor. Biol..

[5] Parker G.A., Drickamer L., Dewsbury D.A. (2010). Reflections before dusk. Leaders in Animal Behavior: The Second Generation.

[6] Parker G.A., Dugatkin L.A. (2001). Golden flies, sunlit meadows: A tribute to the yellow dungfly. Model Systems in Behavioural Ecology: Integrating Conceptual, Theoretical, and Empirical Approaches.

[7] Williams G.C. (1966). Adaptation and Natural Selection: A Critique of Some Current Evolutionary Thought.

[8] Parker G.A., Geoff A. (2007). Parker. Curr. Biol..

[9] Wynne-Edwards V.C. (1962). Animal Dispersion in Relation to Social Behaviour.

[10] Baker R.R., Shackelford T.K., Hansen R.D. (2015). Foreword. The Evolution of Sexuality.

[11] Dawkins R. (1976). The Selfish Gene.

[12] Lehmann L., Keller L., West S., Roze D. (2007). Group selection and kin selection: Two concepts but one process. Proc. Natl. Acad. Sci. USA.

[13] Lehtonen J. (2020). The Price equation and the unity of social evolution theory. Philos. Trans. R. Soc. B Biol. Sci..

[14] Gardner A. (2020). Price’s equation made clear. Philos. Trans. R. Soc. B Biol. Sci..

[15] Baker R.R. (1978). The Evolutionary Ecology of Animal Migration.

[16] Baker R.R., Bellis M.A. (1995). Human Sperm Competition: Copulation, Masturbation and Infidelity.

[17] Baker R.R., Shackelford T.K., Weekes-Shackelford V.A. (2018). Robin Baker and Mark Bellis: Pioneers of research on human sperm competition. Encyclopedia of Evolutionary Psychological Science.

[18] Baker R.R. (2011). Evolutionary psychology and the shaping of the novel Primal. Evol. Psychol..

[19] Baker R. (2013). Sexual whodunits and evolutionary psychology: The shaping of three novels. Evol. Psychol..

[20] Parker G.A. (2010). In celebration of questions, past, present and future. Social Behaviour: Genes, Ecology and Evolution, Székely, T., Moore, A.J., Komdeur, J., Eds..

[21] Parker G.A. (1970). The reproductive behaviour and the nature of sexual selection in *Scatophaga stercoraria* L. (Diptera: Scatophagidae) II. The fertilization rate and the spatial and temporal relationships of each sex around the site of mating and oviposition. J. Anim. Ecol..

[22] Parker G.A. (1974). The reproductive behavior and nature of sexual selection in *Scatophaga stercoraria* L (Diptera-Scatophagidae). IX. Spatial distribution of fertilization rates and evolution of male search strategy within the reproductive area. Evolution.

[23] Parker G.A., Blum M.S., Blum N.A. (1979). Sexual selection and sexual conflict. Sexual Selection and Reproductive Competition in Insects.

[24] Parker G.A., Hardy I.C.W., Briffa M. (2013). Foreword: A personal history of the development of animal contest theory and its role in the 1970s. Animal Contests.

[25] Parker G.A. (1970). The reproductive behaviour and the nature of sexual selection in *Scatophaga stercoraria* L. (Diptera: Scatophagidae) IV. Epigamic recognition and competition between males for the possession of females. Behaviour.

[26] Parker G.A. (1970). Sperm competition and its evolutionary effect on copula duration in the fly *Scatophaga stercoraria*. J. Insect. Physiol..

[27] Parker G.A. (1970). The reproductive behavior and the nature of sexual selection in *Scatophaga stercoraria* L. (Diptera: Scatophagidae). VII. The origin and evolution of the passive phase. Evolution.

[28] Parker G.A., Simmons L.W., Ward P.I. (1993). Optimal copula duration in dungflies: Effects of frequency dependence and female mating status. Behav Ecol. Sociobiol..

[29] Simmons L.W., Parker G.A., Hosken D.J. (2020). Evolutionary insight from a humble fly: Sperm competition and the yellow dungfly. Philos. Trans. R. Soc. Lond. B Biol. Sci..

[30] Darwin C.R. (1871). The Descent of Man, and Selection in Relation to Sex.

[31] Bateman A.J. (1948). Intra-sexual selection in *Drosophila*. Heredity.

[32] Jacobs M.E. (1955). Studies on territorialism and sexual selection in dragonflies. Ecology.

[33] Parker G.A., Lucas J.R., Simmons L.W. (2006). Behavioural ecology: Natural history as science. Essays in Animal Behaviour: Celebrating 50 Years of Animal Behaviour.

[34] Darwin C.R. (1859). On the Origin of Species by Means of Natural Selection.

[35] Parker G.A., Maynard Smith J. (1990). Optimality theory in evolutionary biology. Nature.

[36] Maynard Smith J. (1978). Optimization theory in evolution. Annu. Rev. Ecol. Syst..

[37] Gowaty P.A., Hubbell S.P. (2005). Chance, time allocation, and the evolution of adaptively flexible sex role behavior. Integr. Comp. Biol..

[38] Trivers R.L., Campbell B. (1972). Parental investment and sexual selection. Sexual Selection and the Descent of Man, 1871–1971.

[39] Maynard Smith J., Price G.R. (1973). The Logic of Animal Conflict. Nature.

[40] Andersson M. (1994). Sexual Selection.

[41] Wallace A.R. (1889). Darwinism: An Exposition of the Theory of Natural Selection with Some of its Applications.

[42] Fisher R.A. (1930). The Genetical Theory of Natural Selection.

[43] O’Donald P. (1962). The theory of sexual selection. Heredity.

[44] Huxley J. (1938). Darwin’s theory of sexual selection and the data subsumed by it, in the light of recent research. Am. Nat..

[45] Huxley J., de Beer G.R. (1938). The present standing of the theory of sexual selection. Evolution: Essays on Aspects of Evolutionary Biology.

[46] Roughgarden J., Hoquet T. (2015). Sexual Selection: Is Anything Left?. Current Perspectives on Sexual Selection: What’s left after Darwin?.

[47] Huxley J. (1942). Evolution: The Modern Synthesis.

[48] Mayr E., Campbell B. (1972). Sexual selection and natural selection. Sexual Selection and the Descent of Man, 1871–1971.

[49] Hoquet T. (2020). Bateman (1948): Rise and fall of a paradigm?. Anim. Behav..

[50] Parker G.A., Smith J.L. (1975). Sperm competition and the evolution of the precopulatory passive phase behaviour in Locusta migratoria migratorioides. J. Entomol. Ser. A Gen. Entomol..

[51] Waage J.K. (1979). Dual function of the damselfly penis: Sperm removal and transfer. Science.

[52] Smith R.L. (1979). Repeated copulation and sperm precedence: Paternity assurance for a male brooding water bug. Science.

[53] Sivinski J.L. (1980). Sexual selection and insect sperm. Fla. Entomol..

[54] Lloyd J.E. (1979). Mating behavior and natural selection. Fla. Entomol..

[55] Smith R.L. (1984). Sperm Competition and the Evolution of Animal Mating Systems.

[56] Thornhill R. (1983). Cryptic female choice and its implications in the scorpionfly *Harpobittacus nigriceps*. Am. Nat..

[57] Thornhill R., Alcock J. (1983). The Evolution of Insect Mating Systems.

[58] Simmons L.W. (1986). Female choice in the field cricket Gryllus bimaculatus (De Geer). Anim. Behav..

[59] Eberhard W.G. (1996). Female Control: Sexual Selection by Cryptic Female Choice.

[60] Parker G.A. (1982). Why are there so many tiny sperm—Sperm competition and the maintenance of two sexes. J. Theor. Biol..

[61] Charnov E.L. (1982). The Theory of Sex Allocation.

[62] Parker G.A. (1990). Sperm competition games—Raffles and roles. Proc. R. Soc. Lond. B Biol. Sci..

[63] Parker G.A. (1990). Sperm competition games—Sneaks and extra-pair copulations. Proc. R. Soc. Lond. B Biol. Sci..

[64] Parker G.A., Birkhead T.R., Møller A.P. (1998). Sperm competition and the evolution of ejaculates: Towards a theory base. Sperm Competition and Sexual Selection.

[65] Parker G.A., Pizzari T. (2010). Sperm competition and ejaculate economics. Biol. Rev..

[66] Birkhead T.R. (2000). Promiscuity.

[67] Birkhead T.R., Møller A.P. (1992). Sperm Competition in Birds: Evolutionary Causes and Consequences.

[68] Birkhead T.R., Hosken D.J., Pitnick S. (2009). Sperm Biology: An Evolutionary Perspective.

[69] Simmons L.W. (2001). Sperm Competition and Its Evolutionary Consequences in the Insects.

[70] Parker G.A. (2020). Conceptual developments in sperm competition: A very brief synopsis. Philos. Trans. R. Soc. Lond. B Biol. Sci..

[71] Simmons L.W., Fitzpatrick J.L. (2012). Sperm wars and the evolution of male fertility. Reproduction.

[72] Lüpold S., de Boer R.A., Evans J., Tomkins J., Fitzpatrick J.L. (2020). How sperm competition shapes the evolution of testes and sperm: A meta-analysis. Philos. Trans. R. Soc. Lond. B Biol. Sci..

[73] Parker G.A. (2016). The evolution of expenditure on testes. J. Zool..

[74] Kelly C.D., Jennions M.D. (2011). Sexual selection and sperm quantity: Meta-analyses of strategic ejaculation. Biol. Rev..

[75] DelBarco-Trillo J. (2011). Adjustment of sperm allocation under high risk of sperm competition across taxa: A meta-analysis. J. Evol. Biol..

[76] Poiani A. (2006). Complexity of seminal fluid: A review. Behav. Ecol. Sociobiol..

[77] Avila F.W., Sirot L.K., LaFlamme B.A., Rubinstein C.D., Wolfner M.F. (2011). Insect Seminal Fluid Proteins: Identification and Function. Annu. Rev. Entomol..

[78] Sirot L.K., Wong A., Chapman T., Wolfner M.F. (2015). Sexual conflict and seminal fluid proteins: A dynamic landscape of sexual interactions. Cold Spring Harb. Perspect. Biol..

[79] Wigby S., Brown N., Allen S., Misra S., Sitnik J., Sepil I., Clark A., Wolfner M. (2020). The *Drosophila* seminal proteome and its role in postcopulatory sexual selection. Philos. Trans. R. Soc. Lond. B Biol. Sci..

[80] Ramm S.A. (2020). Seminal fluid and accessory male investment in sperm competition. Philos. Trans. R. Soc. Lond. B Biol. Sci..

[81] Snook R.R. (2005). Sperm in competition: Not playing by the numbers. Trends Ecol. Evol..

[82] Pitnick S., Hosken D.J., Westneat D.F., Fox C.W. (2010). Postcopulatory sexual selection. Evolutionary Behavioral Ecology.

[83] Pitnick S., Wolfner M.F., Dorus S. (2020). Post-ejaculatory modifications to sperm (PEMS). Biol. Rev..

[84] Lüpold S., Pitnick S. (2018). Sperm form and function: What do we know about the role of sexual selection?. Reproduction.

[85] Parker G.A. (2014). The sexual cascade and the rise of pre-ejaculatory (Darwinian) sexual selection, sex roles, and sexual conflict. Cold Spring Harb. Perspect. Biol..

[86] Parker G.A., Pizzari T. (2015). Sexual selection: The logical imperative. Current Perspectives on Sexual Selection.

[87] Kalmus H. (1932). Über den Erhaltungswet den phenotypishen (morphologishen) Anisogamie und die Entstehung der ersten Geshlectsuntershiede. Biol. Zent..

[88] Kalmus H., Smith C.A.B. (1960). Evolutionary origin of sexual differentiation and the sex-ratio. Nature.

[89] Scudo F.M. (1967). The adaptive value of sexual dimorphism. I. Anisogamy. Evolution.

[90] Williams G.C., Mitton J.B. (1973). Why reproduce sexually?. J. Theor. Biol..

[91] Williams G.C. (1975). Sex and Evolution.

[92] Baker R.R., Parker G.A. (1973). The origin and evolution of sexual reproduction up to the evolution of the male-female phenomenon. Acta Biotheor..

[93] Lessells C.M., Snook R.R., Hosken D.J., Birkhead T.R., Hosken D.J., Pitnick S. (2009). 2—The evolutionary origin and maintenance of sperm: Selection for a small, motile gamete mating type. Sperm Biology.

[94] Maynard Smith J. (1978). The Evolution of Sex.

[95] Maynard Smith J. (1982). Evolution and the Theory of Games.

[96] Bell G. (1978). The evolution of anisogamy. J. Theor. Biol..

[97] Charlesworth B. (1978). The population genetics of anisogamy. J. Theor. Biol..

[98] Parker G.A. (1978). Selection on non-random fusion of gametes during the evolution of anisogamy. J. Theor. Biol..

[99] Cox P.A., Sethian J.A. (1984). Search, encounter rates, and the evolution of anisogamy. Proc. Natl. Acad. Sci. USA.

[100] Randerson J.P., Hurst L.D. (2001). The uncertain evolution of the sexes. Trends. Ecol. Evol..

[101] Bulmer M.G., Luttikhuizen P.C., Parker G.A. (2002). Survival and anisogamy. Trends Ecol. Evol..

[102] Bulmer M.G., Parker G.A. (2002). The evolution of anisogamy: A game-theoretic approach. Proc. R. Soc. B-Biol. Sci..

[103] Togashi T., Cox P.A. (2011). The Evolution of Anisogamy: A Fundamental Phenomenon Underlying Sexual Selection.

[104] Parker G.A., Togashi T., Cox P.A. (2011). The origin and maintenance of two sexes (anisogamy), and their gamete sizes by gamete competition. The Evolution of Anisogamy: A Fundamental Phenomenon Underlying Sexual Selection.

[105] Levitan D.R. (1996). Effects of gamete traits on fertilization in the sea and the evolution of sexual dimorphism. Nature.

[106] Iyer P., Roughgarden J. (2008). Gametic conflict versus contact in the evolution of anisogamy. Popul Biol.

[107] Yang J.-N. (2010). Cooperation and the evolution of anisogamy. J. Theor. Biol..

[108] Lehtonen J., Kokko H. (2011). Two roads to two sexes: Unifying gamete competition and gamete limitation in a single model of anisogamy evolution. Behav. Ecol. Sociobiol..

[109] Parker G.A., Lehtonen J. (2014). Gamete evolution and sperm numbers: Sperm competition versus sperm limitation. Proc. R. Soc. B-Biol. Sci..

[110] Lehtonen J., Parker G.A. (2019). Evolution of the two sexes under internal fertilization and alternative evolutionary pathways. Am. Nat..

[111] Randerson J.P., Hurst L.D. (2001). A comparative test of a theory for the evolution of anisogamy. Proc. R. Soc. Lond. Ser. B Biol. Sci..

[112] Hanschen E.R., Herron M.D., Wiens J.J., Nozaki H., Michod R.E. (2018). Multicellularity drives the evolution of sexual traits. Am. Nat..

[113] da Silva J. (2018). The evolution of sexes: A specific test of the disruptive selection theory. Ecol. Evol..

[114] Bell G. (1982). The Masterpiece of Nature: The Evolution and Genetics of Sexuality.

[115] da Silva J., Drysdale V.L. (2018). Isogamy in large and complex volvocine algae is consistent with the gamete competition theory of the evolution of anisogamy. Proc. R. Soc. B Biol. Sci..

[116] Biological Reviews, 200th Anniversary Collection. https://onlinelibrary.wiley.com/page/journal/1469185x/homepage/200th-anniversary-collection.

[117] Behavioural Ecology, Collection: Sperm Competition and its Evolutionary Consequences, Virtual Issues. https://academic.oup.com/beheco/pages/sperm-competition.

[118] Philosophical Transactions of the Royal Society B, Theme Issue: Fifty Years of Sperm Competition. https://royalsocietypublishing.org/toc/rstb/375/1813.

[119] Cells, Special Issue: Origin and Evolution of Sperm Cells—An Issue in Honor of Geoff A. Parker. https://www.mdpi.com/journal/cells/special_issues/honorary_sperm.

