# Supplementary material for: How Soon Hath Time… A History of Two “Seminal” Publications
Source: Cells. 2021 Feb 1;10(2):287. doi: 10.3390/cells10020287 (PMC7912719; doi:10.3390/cells10020287)
Supplement: Supplementary file 1 [file cells-10-00287-s001.pdf]

File S1

Hoquet's critique of Bateman citation in the 1970 paper

Thierry Hoquet's recent (2020) article [1] on Bateman's classic 1948 paper [2] states that:

> "….Geoff Parker (1970) cited Bateman in regard to his interest in the natural history of sperm competition: i.e. mechanisms of insemination, mixture of sperm between males, 'selective fertilization' (predominance of sperm from one genotype during fertilization), and also credited Bateman (1948) for confirming 'that the predominance of the second male is related to the interval (and therefore amount of oviposition) between matings' (Parker, 1970, p. 530). But this is quite an unexpected and surprising lesson for anyone to draw from Bateman's paper because Bateman never monitored the mating behaviour of his experimental flies and, consequently, could not (and did not) provide any information about 'interval between matings'."

It is certainly true that Bateman did not record details of mating behaviour. My citation related to his conclusions about sperm predominance in relation to flies with specific genetic markers present together in vials, and the timing of their matings. So Bateman did provide information about 'interval between matings'. He could deduce when a given male had mated with a given female from certain of his experiments in which he transferred the same batches of flies to different bottles each day. His paternity analyses of the genetic markers in progeny then gave information about the day on which a given mating had occurred.

In section A below I give details of what Bateman wrote about this issue. Next, in section B, I give details from my records of how my citation of Bateman arose and how it became modified in revision of the manuscript.

A. Bateman's (1948) paper [2]

p. 353. METHODS, last paragraph. Bateman discusses the fact that three previous authors claim that

> "…. when a female is mated twice, the second batch of sperm supersedes the first which only reappears in the progeny when the two matings occurred in quick succession for no progeny from the first mating to appear."

p. 354, top. Bateman then states that

> "However, Lobashov (1939) on the other hand claimed to get complete mixture of sperm. Some of the data [from what follows, it is evident that this is Bateman's data] enable one to assess the likelihood that replacement of one kind of sperm by another might interfere with the results."

He then goes on to point out that his experiments (series 5) enabled him to determine whether this would be a problem:

> "In series 5 (see table 3) the mated flies were transferred each day for 4 days to new bottles. In this way it was possible to observe whether a female producing progeny derived from the sperm of two males utilized the two batches of sperm separately or together."

He follows this with a (non-numbered) Table showing his results for experiments where males were transferred to different bottles, and then concludes explicitly that:

> "Evidently if the second insemination occurs soon after the first there is complete mixing of sperm, but as the interval of time between inseminations increases the second insemination becomes more likely to supersede the first."

B. My manuscripts and publication of the 1970 *Biol. Rev.* article [3]

I still retain the original manuscripts and correspondence relating to my 1970 paper, which reveal how my citation arose and how it was modified during the review process:

1. *First notes.* These are handwritten, in black biro, written before I rough-typed the first draft of the manuscript to hand to secretaries for final typing for submission. A note is written at the top of the relevant page in pencil, reminding me to add:

> "Bateman (1948) cleared up dispar. by showing that mixing on first day not later"

The disparity ("dispar.") related to the differences between authors in the pattern of sperm competition. (I gave more sources for *Drosophila* sperm competition than Bateman).

2. *First submitted draft.* This was the first typed manuscript submitted to the *Biol. Rev.* editorial office, 18th May 1970. Here the pencil note had been amplified as follows:

> "Using genetically marked individuals Bateman (1948) confirmed that if the second mating occurs soon after the first there is complete mixing of sperm used for fertilization, but with increased interval between matings the second insemination tends to predominate over the first."

3. *Revised manuscript.* The manuscript was too long, and the editor required it to be shortened by several thousand words (see main text). This was achieved mainly by reducing the experimental

evidence section in which the *Drosophila* data were reviewed. In the revised typescript, this sentence was much reduced, and had become somewhat cryptic:

> "Bateman (1948) confirmed that the predominance of the second male is related to the interval (and therefore the amount of oviposition) between matings."

This is the citation as published in the *Biol. Rev.* 1970 publication, albeit in much-reduced form from what I had originally intended. Though I had added my own (I believe fair) conclusion about the amount of oviposition, it reports what Bateman claimed to have found from his experiments, which did not involve observing behaviour, but rather, by making deductions about behaviour from his genetic marking experiments. It is hardly, therefore, "quite an unexpected and surprising lesson for anyone to draw from Bateman's paper", as Hoquet claims [1].

Comment on the underlying science

In 1976 we conducted our own experiments on *Drosophila melanogaster* in which matings were observed directly [4], something I have always advocated for sperm competition studies. They suggested that though the predominance of the second male to mate does increase (as Bateman claimed) with time after the first mating; there was high sperm predominance (83%) by the second male even when the second mating occurred the day after the first. Different strains of *Drosophila* are likely to have different responses.

1. Hoquet, T. Bateman (1948): rise and fall of a paradigm? *Anim Behav* **2020**, *164*, 223-231, doi:10.1016/j.anbehav.2019.12.008.
2. Bateman, A.J. Intra-sexual selection in *Drosophila*. *Heredity* **1948**, *2*, 349-368.
3. Parker, G.A. Sperm competition and its evolutionary consequences in the insects. *Biological Reviews* **1970**, *45*, 525-567.
4. Boorman, E.; Parker, G.A. Sperm (ejaculate) competition in *Drosophila melanogaster*, and reproductive value of females to males in relation to female age and mating status. *Ecol Entomol* **1976**, *1*, 145-155, doi:10.1111/j.1365-2311.1976.tb01217.x.
